# Practical utility of general practice data capture and spatial analysis for understanding COPD and asthma

**DOI:** 10.1186/s12913-018-3714-5

**Published:** 2018-11-26

**Authors:** T. Niyonsenga, N. T. Coffee, P. Del Fante, S. B. Høj, M. Daniel

**Affiliations:** 1 Centre for Research and Action in Public Health, Health Research Institute, Faculty of Health, University of Canberra, Canberra, Australian Capital Territory Australia; 2 Healthfirst Network, Adelaide, South Australia Australia; 3 Centre de Recherche du Centre Hospitalier de l’Université de Montréal (CRCHUM), Montreal, Quebec Canada; 4 Centre for Population Health Research, School of Health Sciences, University of South Australia, Adelaide, South Australia Australia; 5 Department of Medicine, St Vincent’s Hospital, The University of Melbourne, Melbourne, Victoria Australia

**Keywords:** COPD and asthma, General practice capture data, Chronic disease management, Improved research quality data, Spatial analysis

## Abstract

**Background:**

General practice-based (GP) healthcare data have promise, when systematically collected, to support estimating local rates of chronic obstructive pulmonary disease (COPD) and asthma, variations in burden of disease, risk factors and comorbid conditions, and disease management and quality of care. The use of GP information systems for health improvement has been limited, however, in the scope and quality of data. This study assessed the practical utility of de-identified clinical databases for estimating local rates of COPD and asthma. We compared COPD and asthma rates to national benchmarks, examined health related risk factors and co-morbidities as correlates of COPD and asthma, and assessed spatial patterns in prevalence estimates at the small-area level.

**Methods:**

Data were extracted from five GP databases in western Adelaide, South Australia, for active patients residing in the region between 2012 and 2014. Prevalence estimates were computed at the statistical area 1 (SA1) spatial unit level using the empirical Bayes estimation approach. Descriptive analyses included summary statistics, spatial indices and mapping of geographic patterns. Bivariate associations were assessed, and disease profiles investigated to ascertain multi-morbidities. Multilevel logistic regression models were fitted, accounting for individual covariates including the number of comorbid conditions to assess the influence of area-level socio-economic status (SES).

**Results:**

For 33,725 active patients, prevalence estimates were 3.4% for COPD and 10.3% for asthma, 0.8% higher and 0.5% lower for COPD and asthma, respectively, against 2014–15 National Health Survey (NHS) benchmarks. Age-specific comparisons showed discrepancies for COPD in the ‘64 years or less’ and ‘age 65 and up’ age groups, and for asthma in the ‘15–25 years’ and ‘75 years and up’ age groups. Analyses confirmed associations with individual-level factors, co-morbid conditions, and area-level SES. Geographic aggregation was seen for COPD and asthma, with clustering around GP clinics and health care centres. Spatial patterns were inversely related to area-level SES.

**Conclusion:**

GP-based data capture and analysis has a clear potential to support research for improved patient outcomes for COPD and asthma via knowledge of geographic variability and its correlates, and how local prevalence estimates differ from NHS benchmarks for vulnerable age-groups.

## Background

Chronic Obstructive Pulmonary Disease (COPD) is a progressive condition that affects the respiratory and circulatory systems, and is most often caused by the inhalation of noxious particulates or gases which in turn stimulate an abnormal inflammatory response from the airways and the lungs [1–4]. Asthma, on the other hand, is a common chronic disorder of the airways which is complex with variable and recurring symptoms of airflow obstruction, bronchial hyper-responsiveness, and an underlying inflammation [5]. It is characterised by reversible (or partly reversible) intermittent or chronic airway inflammation and respiratory symptoms such as wheezing, shortness of breath, chest tightness, and coughing which vary over time and in intensity [1–4]. Both COPD and asthma can co-exist in the same patient posing diagnostic and therapeutic challenges. This correspondence has been recognized by a joint committee of the Global Initiative for Chronic Obstructive Lung Disease (GOLD) and the Global Initiative for Asthma (GINA) as the “asthma-COPD overlap” (ACO) syndrome. While there remains controversy as to whether this overlap constitutes a syndrome [2–4, 6], important features distinguish typical COPD from typical asthma. For example, people with COPD continue to lose lung function despite taking medication, not a common feature of asthma [7]. People with COPD and/or asthma rate their health worse than people without these conditions [7, 8]. In the later stages of COPD, the inflammatory response interferes with normal breathing patterns and exercise tolerance, resulting in poor quality of life and dependence on community and carer support [8, 9]. Disease management is difficult and hospitalisation for episodes of acute illness is common, with longer duration of admissions compared to other chronic conditions [10]. Despite declining mortality rates for asthma and COPD in Australia [11], the asthma death rate remains high compared with many other countries while COPD is a leading cause of death both domestically and internationally [12]. Both COPD and asthma are potentially preventable hospitalisation conditions, with COPD the second leading cause of avoidable hospital admissions [13].

Geographically varying socio-economic correlates and risk factors exist for COPD and asthma, including genetic disposition, behavioural and environmental factors [1, 14, 15]. Most of the people develop COPD through environmental exposures to air pollutants and deleterious gases, particularly, the exposure to tobacco smoke including second hand smoke. Other significant and spatially varying environmental sources include emissions from local industries, proximity to major roads and heavy traffic, occupational exposures to dust, particularly among workers in coal mining or livestock farming [1, 14]. Early diagnosis and secondary prevention can be achieved through screening and close monitoring by general practitioners (GPs) [16–18]. But GPs often do not have the capacity to screen for patients at risk and, consequently, most of cases are detected in the latter stages of the disease when symptoms become pronounced and/or begin to impair patient’s quality of life [19, 20]. This situation presents an opportunity for improvement as, in Australia, 75% of all medical consultations take place in GP’s offices, and more than 85% of the population access a GP each year [21]. Providing GPs with the knowledge of what the rates of COPD and asthma are in their local area, the levels of risk in the local community, and the characteristics of patient catchment areas could enable GPs to provide improved patient care [22–25].

COPD and asthma are exemplar conditions by which to illustrate how GP data capture and analysis can aid in understanding the local features of important diseases and pertinent care. GP-based data can offer an important source of real-world information on the populations accessing practices, and local-area living conditions. Spatial analyses of GP-based data can assist to better understand geographic distributions of patients’ health status and outcomes, and inform local health services improvements. Thus far, however, the use of GP-based clinical information systems for data collection and health improvement has been limited in both extent and quality. Models such as the Practice Health Atlas (PHA) have been used to develop a professional culture around quality health data [26] and platforms such as the Bettering the Evaluation and Care of Health (BEACH) and the Melbourne East Monash General Practice Database (MAGNET) have been developed to provide unique and high-quality GP research databases [27, 28]. These models should be extended to the entire Australian GP body to unlock the potential of high-quality GP data for high-quality research contributing to improved local and global patients’ health outcomes [24, 26–28].

**Fig. 1:**
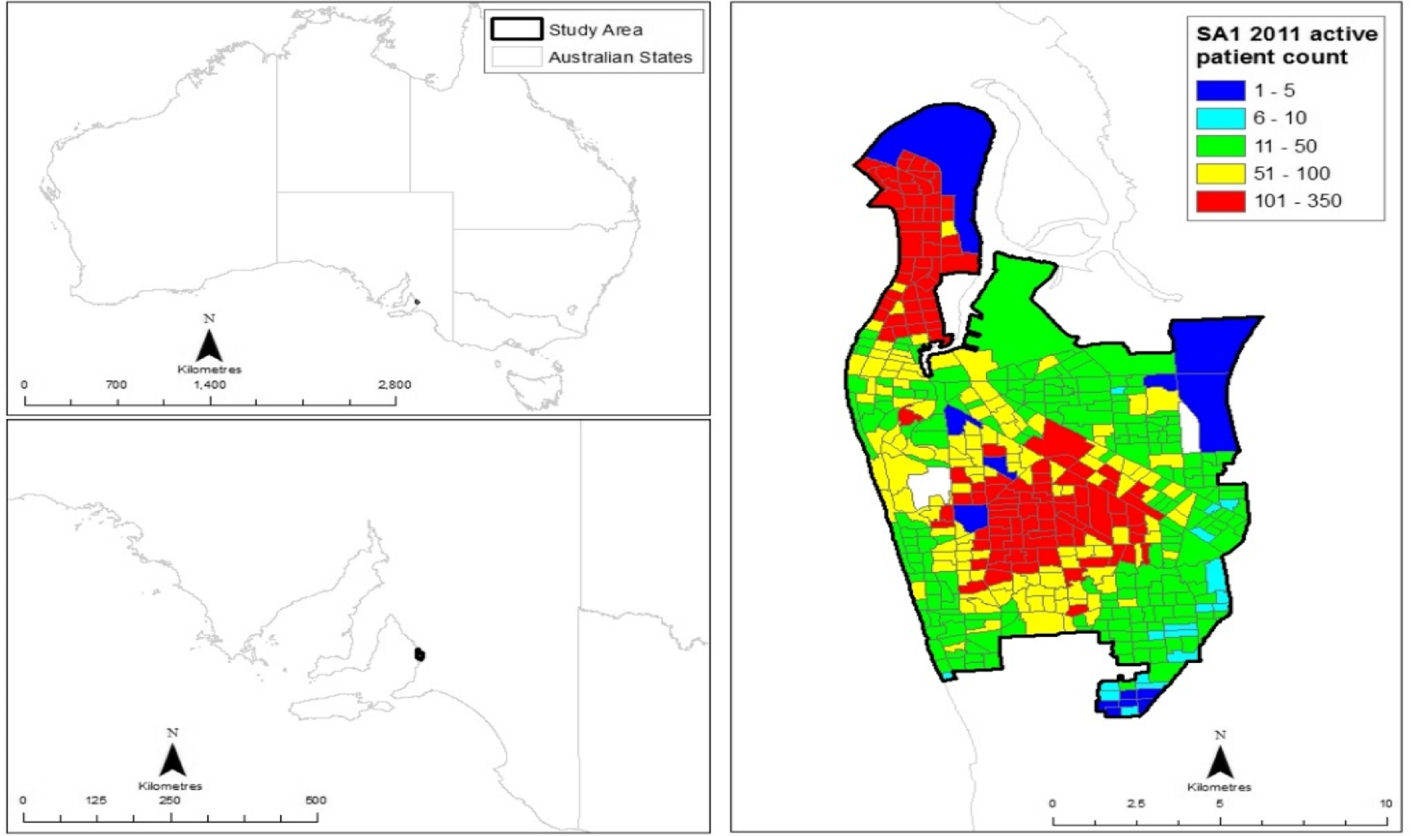
Delimitation of the study area and SA1 counts of active patients

In 2012–13, a Medicare Local-based intervention program to improve GP data quality and reduce GP-based COPD under-diagnosis was implemented in the western region of Adelaide, South Australia (Fig. 1). Baseline data from five large practices in the area indicated that, compared to the national benchmark, there was a much lower overall rate of COPD (2% of GP population undiagnosed) [29]. Within 6 months of the program, the proportion of patients with a diagnosis of COPD increased on average by 20% [29]. The purpose of the current study was to use GP-based de-identified clinical data from five practices who participated in the above intervention program with aims to address: 1) the practical use of GP-based data for estimating localized COPD and asthma spatial prevalence rates, comparing GP-based rates with national rates; 2) the use of GP-based data to examine risk factors and comorbid conditions associated with COPD and asthma; and 3) whether spatial clustering exists in COPD and asthma prevalence rates, and if so, whether such clustering varies according to area-level socio-economic status (SES).

## Methods

### Source of data

Data were extracted from five “sentinel” GP practices participating in the Medicare Local GP-based data improvement program in the western area of Adelaide, South Australia, including the LeFevre Peninsula and its closest surrounding areas. Figure 1 displays the study region according to the Australian Bureau of Statistics (ABS) census unit, statistical area 1 (SA1) [30]. “Sentinel” GP practices were chosen for the availability of higher-quality GP data and the need to understand population factors related to chronic disease. Analyses were restricted to “active” patients living within the study area and seen by their GP between 2012 and 2014. An “active” patient was defined per the Royal Australian College of General Practitioners (RACGP) criterion as one who attended the practice three times or more within the past two years [31].

GP clinical data from this region have previously been used to examine spatial variation of cardiovascular disease at small geographic area levels and to estimate community level prevalence of undiagnosed diabetes [32, 33]. There were 486 SA1s in the study area. Table 1 below presents the five practices included in the analysis and their respective records of total and active patients within the study area. There were 33,725 active patients who visited GP practices with 3 progress notes recorded. The de-identified GP clinical records included the risk factors age, sex, smoking status, Aboriginal or Torres Strait Islander status (ATSI), and marital status. Respiratory health conditions under study (outcome variables) were COPD, asthma, and ACO. Recorded comorbidities included type 2 diabetes, heart failure, stroke, peripheral vascular disease, mental disorder, dementia and osteoarthritis/osteoporosis. Variables relating to respiratory health conditions, risk factors and comorbidities were coded in accordance with value domain attributes listed on the Australian Institute of Health and Welfare (AIHW) Metadata Online Registry (Meteor). Respiratory health conditions were classified as “active” if active at the time of the extract; “inactive” and “never diagnosed” conditions were combined and classified as the absence of a condition. The classification of active COPD was satisfied by a record of either active respiratory COPD or active chronic obstructive airways disease. Finally, the 2011 census index of relative socio-economic disadvantage (IRSD), one of the socio-economic index of areas (SEIFA) variants, was obtained from the Australian Bureau of Statistics (ABS) [34].

**Table 1:** Counts of total patients and active patients recorded by each practice in the initial database and restricted to residents of the study area

	Initial database records		Restricted study area	
Practice ID	Total patients	Active patients	Total patients	Active patients
Practice 1	25,062	13,655	18,958	12,032
Practice 2	23,161	9858	17,551	8004
Practice 3	24,838	9349	15,548	7085
Practice 4	8301	4690	6863	4140
Practice 5	8098	2919	6198	2464
Total	89,460	40,471	65,118	33,725

The 2014–15 National Health Survey (NHS), the most recent in a series of Australia-wide health surveys conducted by the ABS, provided information on national prevalence estimates of chronic respiratory conditions by pre-defined age groups [7]. The NHS was designed to collect a range of information about the health of Australians, including: prevalence of long-term health conditions; health risk factors such as smoking, overweight and obesity, alcohol consumption and exercise; use of health services such as consultations with health practitioners and actions people have recently taken for their health; and demographic and socio-economic characteristics.

Both prevalence rates and standardised prevalence ratios (SPR) of COPD and asthma were calculated by SA1 geographic areas. Prevalence estimates were adjusted for the number of active patients in each SA1 using the empirical Bayes estimation approach [35]. SPRs were computed as the ratio of observed to expected number of cases in each area, where the expected number of cases was determined according to the age distribution of patients within each SA1. Specifically, expected levels of risk were attributed to patients based on their age groups: 0–14, 15–24, 25–34, 35–44, 45–54, 55–64, 65–74, 75–84 and 85+, and associated nationwide estimates of asthma and COPD risks for these age groups from 2014 to 15 NHS data [7]. Expected risk was then summed across all patients in the SA1 to determine the expected number of cases. These SPRs were purposively calculated to highlight under- or over-diagnosed areas. Area-level prevalence and SPRs estimates were mapped to highlight the geographic variation of COPD and asthma.

### Descriptive and inferential analyses

Descriptive analyses consisted of summary statistics, spatial indices (for spatial clustering) and mapping of geographical patterns. Summary statistics included descriptive statistics pertaining to: (1) the prevalence of diagnosed respiratory health conditions (COPD, asthma, and ACO) among active patients in the study area; and (2) the prevalence of these diagnosed respiratory conditions across distinct age groups, benchmarked against NHS data for the years 2014–15 [7]. Bivariate associations between these diagnosed respiratory conditions and individual risk factors and comorbidities were assessed, and between-group differences tested for statistical significance using Pearson’s chi-square test or Fisher’s exact test where observed cell numbers were small. Chronic disease profiles were investigated for multi-morbidities and a multi-morbidity matrix that reflected the number and percentage of patients with co-occurring conditions was calculated.

To assess whether area-level SES, measured by SEIFA-IRSD, was related to COPD and asthma prevalence rates, multilevel logistic regression models of active COPD and asthma among patients nested within SA1s were performed (for inferential analysis [36]), accounting for basic individual socio-demographic covariates (age, sex, smoking status, marital status and ATSI) and the number of comorbid conditions. All covariates were categorical, except age which was modelled as a standardized continuous variable. Linear and quadratic age terms were incorporated in the models.

Distributions of COPD and asthma cases were mapped and the degree to which cases were clustered spatially (spatial autocorrelation, ‘Hot’ and ‘cold’ spots) was assessed using indices of spatial associations. Global Moran’s I (for spatial autocorrelation) and local Getis-Ord’s Gi* (for hot or cold spots) indices were computed [37, 38]. Bivariate choropleth maps of COPD and asthma prevalence against SEIFA-IRSD were created to visualise the extent of geographic overlap in spatial clustering patterns of each measure at the SA1 level. SEIFA-IRSD and estimated prevalence values were, for each variable, split into quartiles, and then the variables were overlayed to indicate regions of high prevalence/high disadvantage, low prevalence/low disadvantage, etc.

## Results

### Descriptive analysis

Overall observed prevalence estimates were 3.4, 10.3 and 0.9% for COPD, asthma and ACO respectively (Table 2). As expected, COPD was more prevalent in the 55 years and over group (7.8%) while asthma was more prevalent in the up to 34 years group (11.7%). At the practice level, overall prevalence estimates for COPD and asthma were less than national self-reported prevalence benchmarks (2.6 and 10.8% for COPD and asthma respectively) at one (COPD) and three (asthma) of the five practices analysed (Tables 3 and 4). Conversely, more COPD cases than expected were observed at one of the five practices analysed, and more asthma cases than expected at two of the five (Tables 3 and 4). Overall SPR values were 0.92 and 0.96 for COPD and asthma respectively, indicating both COPD and asthma under-diagnosis from GP data.

**Table 2:** Prevalence of active COPD, asthma, and combined asthma-COPD overlap among active patients in the study area

	COPD		Asthma		Overlap (COPD + asthma)		Total (row n)
All active patients	n	%	n	%	n	%	n
	1130	3.4	3457	10.3	317	0.9	33,725
By age group	n	%	n	%	n	%	n
0–14	2	0.0	524	10.9	1	0.0	4789
15–24	0	0.0	474	14.2	0	0.0	3328
25–34	10	0.3	363	10.2	3	0.1	3561
35–44	19	0.5	391	9.4	6	0.1	4180
45–54	82	1.7	432	8.9	24	0.5	4846
55–64	190	4.2	389	8.7	50	1.1	4479
65–74	288	7.5	379	9.9	77	2.0	3846
75–84	359	11.1	364	11.3	103	3.2	3221
85+	180	12.2	141	9.6	53	3.6	1474

**Table 3:** Prevalence and standardised prevalence ratios of COPD by medical practice

	Active patients	Observed cases	Expected cases	Prevalence (=O/A*100%)	SPR (=O/E)
Practice 1	12,032	374	409	3.1	0.91
Practice 2	8004	345	290	4.3	1.19
Practice 3	7085	243	269	3.4	0.90
Practice 4	4140	103	154	2.5	0.67
Practice 5	2464	65	77	2.6	0.84
Total	33,725	1130	1228	3.4	0.92

Note: *SPR* Standardised prevalence ratio

**Table 4:** Prevalence and standardised prevalence ratios of asthma by medical practice

	Active patients	Observed cases	Expected cases	Prevalence (=O/A*100%)	SPR(=O/E)
Practice 1	12,032	1320	1297	11.0	1.02
Practice 2	8004	815	855	10.2	0.95
Practice 3	7085	828	753	11.7	1.10
Practice 4	4140	332	440	8.0	0.76
Practice 5	2464	162	266	6.6	0.61
Total	33,725	3457	3605	10.3	0.96

Note: *SPR* Standardised prevalence ratio

**Fig. 2:**
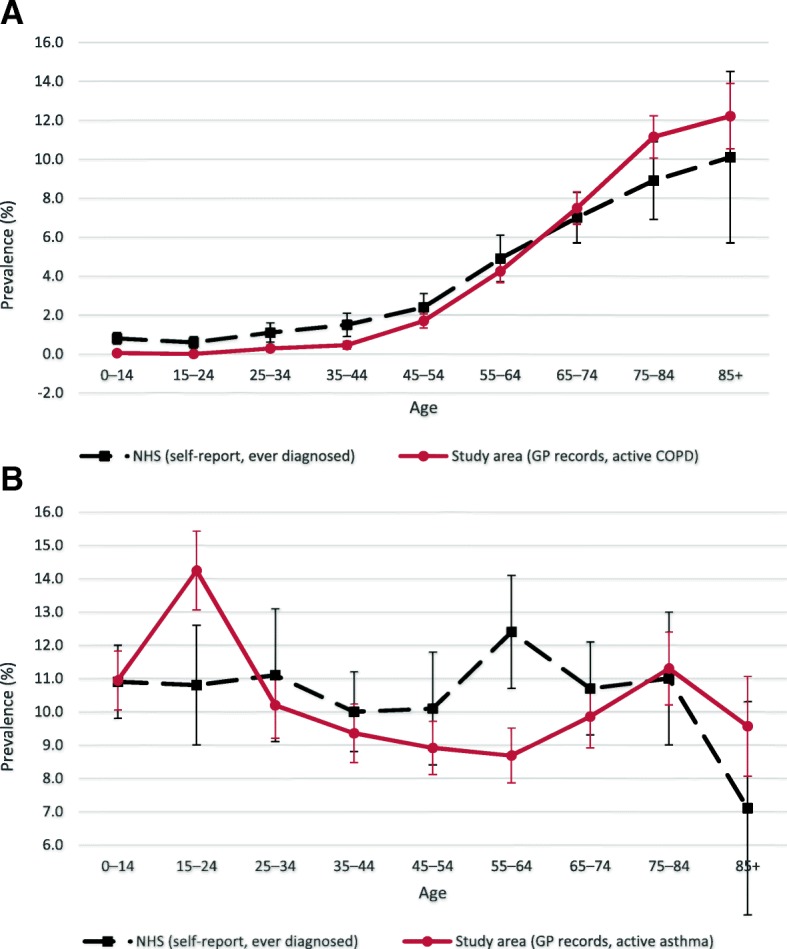
**a** Prevalence of COPD by age group in study area versus NHS statistics with 95% confidence intervals. **b** Prevalence of Asthma by age group in study area versus NHS statistics with 95% confidence intervals

Although data from these “sentinel” GP practices indicated a 0.8% higher overall rate of COPD and a 0.5% lower overall rate of asthma (compared to national benchmarks), prevalence estimates were lower than the benchmark before age 65 (0.6% lower for those aged 15–24, and 1.0% lower for those aged 35–44) but higher than the benchmark for age 65 years and over (0.5, 2.2 and 2.1% higher for the age groups 65–74, 75–84 and 85+) for COPD (Fig. 2a). For asthma, prevalence estimates in those aged 15–24 and 85+ were 3.4 and 2.5% higher, respectively, than the benchmark. On the other hand, asthma under-diagnosis was apparent between age 25 and 74 years, up to 3.7% lower for those aged 55–64 years (Fig. 2b).

### COPD, asthma, risk and/or comorbid conditions

Differences in prevalence estimates for active respiratory conditions for patients defined according to their status on a given risk factor, or the presence/absence of another health condition, highlight the magnitude of association between COPD and asthma with risk factors and comorbid conditions (Table 5). Analyses of COPD generally yielded strong statistically significant associations with known individual-level factors and comorbid conditions (all *p*-values < 0.0001), except for ATSI status. For asthma, prevalence rates were statistically significantly different across levels of individual-level characteristics, except smoking status. Asthma prevalence rates also differed significantly in relation to examined comorbid conditions, except for peripheral vascular disease, stroke and dementia for which associations were statistically non-significant.

**Table 5:** Respiratory conditions within risk factors/comorbidities groups (count & prevalence)

	COPD		Asthma		ACO (COPD + asthma)		Total (row n)
	n	%	n	%	n	%	n
All active patients	1130	**3.4**	3457	**10.3**	317	**0.9**	**33,725**
Risk factors							
Age		****		****		****	**33,724**
Under 55	113	**0.5**	2184	**10.5**	34	**0.2**	20,704
55 to 69	327	**4.9**	595	**9.0**	83	**1.2**	6648
70 to 84	510	**10.4**	537	**11.0**	147	**3.0**	4898
85 and over	180	**12.2**	141	**9.6**	53	**3.6**	1474
Sex		****		****			**33,627**
Male	591	**4.1**	1380	**9.6**	138	**1.0**	14,375
Female	539	**2.8**	2073	**10.8**	179	**0.9**	19,252
Smoking^a^		****				****	**25,688**
Daily/irregular	298	**7.8**	442	**11.6**	63	**1.7**	3807
Ex-smoker	518	**8.9**	660	**11.3**	148	**2.5**	5839
Never smoked	236	**1.5**	1775	**11.1**	85	**0.5**	16,042
Indigenous Australian				**			**15,304**
Yes	15	**5.4**	45	**16.1**	4	**1.4**	280
No	565	**3.8**	1618	**10.8**	185	**1.2**	15,024
Marital status		****		****		****	**9853**
Never married	97	**2.8**	423	**12.1**	29	**0.8**	3496
Married/De facto	221	**4.3**	470	**9.1**	53	**1.0**	5167
Divorced/Separated	41	**7.8**	67	**12.7**	12	**2.3**	526
Widowed	66	**9.9**	92	**13.9**	23	**3.5**	664
Comorbid conditions							
Diabetes (type II)		****		**		****	**33,725**
Yes	184	**7.7**	291	**12.1**	58	**2.4**	2399
No	946	**3.0**	3166	**10.1**	259	**0.8**	31,326
Heart failure		****		****		****	**33,725**
Yes	91	**20.0**	79	**17.3**	35	**7.7**	456
No	1039	**3.1**	3378	**10.2**	282	**0.8**	33,269
Stroke		****				****	**33,725**
Yes	66	**12.0**	68	**12.3**	16	**2.9**	552
No	1064	**3.2**	3389	**10.2**	301	**0.9**	33,173
Peripheral vascular disease		****				**	**33,725**
Yes	37	**23.9**	12	**7.7**	6	**3.9**	155
No	1093	**3.3**	3445	**10.3**	311	**0.9**	33,570
Mental disorder		****		****		****	**33,725**
Yes	310	**5.8**	804	**15.0**	99	**1.8**	5362
No	820	**2.9**	2653	**9.4**	218	**0.8**	28,363
Dementia		****				*	**33,725**
Yes	26	**10.1**	22	**8.6**	7	**2.7**	257
No	1104	**3.3**	3435	**10.3**	310	**0.9**	33,468
Osteoarthritis/ osteoporosis		****		****		****	**33,725**
Yes	550	**9.7**	776	**13.6**	189	**3.3**	5698
No	580	**2.1**	2681	**9.6**	128	**0.5**	28,027

Note: Pearson Chi-square test: * p < 0.05, ** p < 0.01, *** p < 0.001, **** p < 0.0001; Mental disorder: any active anxiety, depression or bipolar disorder; Row totals do not always add to the total number of active patients due to missing data; ^a^Missing data on smoking within active COPD (*n =* 78, 6.9%)*;* asthma (*n* = 580, 17%)*; ACO* (*n =* 21, 6.6%)

Stars (***) inside Table 5 indicate the levels of the Pearson Chi-square test p-values (*p*); *: *p* < 0.05; **: *p* < 0.01; ***: *p* < 0.001; ****: *p* < 0.0001

Within the five GP practices, 50.7% of patients had at least one chronic condition, and 21.1% at least two conditions. For patients with COPD, 83.7% had at least two additional chronic conditions including asthma. For patients with asthma, 59.8% had at least two additional chronic conditions including COPD. As shown in Table 6, prevalent co-occurring chronic conditions among patients with COPD included osteoarthritis/osteoporosis (48.7%), asthma (28.1%) and mental health disorder (27.4%). For co-occurring chronic conditions with asthma, besides COPD-asthma, the most frequent condition was mental health disorder (23.3%) followed by osteoarthritis/osteoporosis (22.4%).

**Table 6:** Percentage of patients with an active condition (rows) that also have an additional active condition (columns)

			*Condition B*								
*Condition A*	*Row n*	*Row %*	*COPD*	*Asthma*	*OA/Osteo*	*Mental*	*Diabetes*	*Stroke*	*Heart fail*	*Dementia*	*PVD*
*COPD*	*1130*	*3.35*	100	28.1	48.7	27.4	16.3	5.84	8.05	2.30	3.27
*Asthma*	*3457*	*10.3*		100	22.4	23.3	8.42	1.97	2.29	0.64	0.35
*OA/Osteo condition*	*5698*	*16.9*			100	28.7	16.5	5.76	4.97	2.49	1.67
*Mental disorder*	*5362*	*15.9*				100	10.3	3.02	2.16	1.90	0.67
*Diabetes type II*	*2399*	*7.11*					100	5.42	4.50	2.25	2.00
*Stroke*	*552*	*1.64*						100	10.3	5.43	2.72
*Heart failure*	*456*	*1.35*							100	6.36	4.39
*Dementia*	*257*	*0.76*								100	2.72
*PVD*	*155*	*0.46*									100
*Overall prevalence of Condition B*			*3.35*	*10.3*	*16.9*	*15.9*	*7.11*	*1.64*	*1.35*	*0.76*	*0.46*

*Note – OA/Osteo condition* osteoarthritis or osteoporosis, *Mental disorder* any active anxiety, depression or bipolar disorder, *PVD* peripheral vascular disease

Among patients aged 55 years and over (of whom 7.8% were diagnosed with COPD, and 9.8% with asthma), 72.2% had at least one other chronic condition and, 84.5% of COPD patients and 86.3% of asthma patients had at least two additional conditions. Prevalent pairs of co-occurrence conditions were asthma-osteoarthritis/osteoporosis (53.7%) and COPD-osteoarthritis/osteoporosis (51.9%), followed by asthma-mental health disorder (29.3%) and COPD-asthma (29.3%) (Table 7).

**Table 7:** Percentage of patients aged 55 years and over with an active condition (rows) that also have an additional active condition (columns)

			*Condition B*								
*Condition A*	*Row n*	*Row %*	*COPD*	*Asthma*	*OA/Osteo*	*Mental*	*Diabetes*	*Stroke*	*Heart fail*	*Dementia*	*PVD*
*COPD*	*1017*	*7.81*	100	27.8	51.9	25.4	17.7	6.49	8.95	2.56	3.54
*Asthma*	*1273*	*9.78*		100	53.7	29.3	19.2	5.26	6.05	1.65	0.86
*OA/Osteo condition*	*5105*	*39.2*			100	27.4	17.7	6.39	5.45	2.76	1.84
*Mental disorder*	*2618*	*20.1*				100	17.6	5.77	4.35	3.86	1.38
*Diabetes type II*	*2074*	*15.9*					100	6.17	5.16	2.60	2.31
*Stroke*	*527*	*4.05*						100	10.8	5.50	2.85
*Heart failure*	*442*	*3.40*							100	6.56	4.52
*Dementia*	*253*	*1.94*								100	2.77
*PVD*	*150*	*1.15*									100
*Overall prevalence of Condition B*			*7.81*	*9.78*	*39.2*	*20.1*	*15.9*	*4.05*	*3.40*	*1.94*	*1.15*

*Note – OA/Osteo condition* osteoarthritis or osteoporosis, *Mental disorder* any active anxiety*,* depression or bipolar disorder, *PVD* peripheral vascular disease

### COPD, asthma and SEIFA - index of relative social disadvantage

Results of multilevel models to assess the effects of area-level SES (SEIFA-IRSD) on COPD and asthma prevalence rates, adjusting for patient characteristics, are presented in Tables 8 and 9. The unadjusted model of COPD and area-level SES relationships exhibited a significant negative gradient from the least (Q4) to the most (Q1) disadvantaged quartile groups. This gradient was also observed in the adjusted model, where the estimated risk of COPD among patients residing in the most disadvantaged SA1s (Q1) was 1.67 times greater than that of patients in least disadvantaged SA1s (OR = 1.670; 95% CI: 1.168–2.387). These patients (from SA1s in Q1) faced also a risk of COPD 1.169 times greater than patients from SA1s in Q2 (OR = 1.169; 95% CI: 0.977–1.398), although this association was at the borderline of statistical significance (Table 8). The adjusted model additionally confirmed the importance of individual-level covariates on the prevalence of COPD, especially the number of comorbid conditions as well as age, sex and smoking status.

**Table 8:** Multilevel logistic regression of active COPD

	*Model 1*					*Model 2*				
Covariates	*Est.*	*SE*	*p*	*[95%*	*CI]*	*Est.*	*SE*	*p*	*[95%*	*CI]*
Within-cluster										
Age	–	–	–	–	–	2.348	0.239	< 0.0001	[1.880	2.816]
Age^2^	–	–	–	–	–	− 0.697	1.121	< 0.0001	[− 0.933	− 0.461]
Male	–	–	–	–	–	0.522	0.077	< 0.0001	[0.372	0.672]
Female	–	–	–	–	–	0.0	–	–	–	–
Smoker	–	–	–	–	–	2.477	0.116	< 0.0001	[2.251	2.704]
Ex-smoker	–	–	–	–	–	1.522	0.093	< 0.0001	[1.340	1.704]
Never smoked	–	–	–	–	–	0.0	–	–	–	–
Indigenous Australian	–	–	–	–	–	0.871	0.341	0.011	[0.203	1.540]
Non-Indigenous Australian	–	–	–	–	–	0.0	–	–	–	–
Unmarried	–	–	–	–	–	0.365	0.126	0.004	[0.118	0.613]
Married	–	–	–	–	–	0.0	–	–	–	–
N-Comorbidities	–	–	–	–	–	1.060	0.029	< 0.0001	[1.004	1.117]
Between-cluster				–	–					
IRSD (Q4)	−1.280	0.171	< 0.0001	[−1.614	−0.945]	− 0.513	0.182	0.005	[−0.870	− 0.155]
IRSD (Q3)	−0.712	0.114	< 0.0001	[−0.936	− 0.489]	− 0.299	0.117	0.010	[− 0.528	− 0.071]
IRSD (Q2)	− 0.358	0.083	< 0.0001	[− 0.520	− 0.196]	− 0.156	0.091	0.088	[− 0.335	0.023]
IRSD (Q1)	0.0	–	–	–	–	0.0	–	–	–	–

Notes – Age: age in years, standardised; Age^2^: Age*Age. Smoker: daily, weekly or irregular smoker; Ex-smoker: does not smoke now, but has smoked ≥ 100 cigarettes (or equivalent) in life time; Never smoked: does not smoke now, and has smoked < 100 cigarettes (or equivalent) in life time; Unmarried: never married, divorced, separated or widowed; Married: registered or de facto; N-Comorbidities: Number of comorbid conditions; IRSD: Index of Relative Social Disadvantage (ABS 2011); Q1-Q4: National quartile of IRSD (determined using cut-points for quartiles within the Australia-wide distribution of scores at SA1 level) where Q1: most disadvantaged, Q4: least disadvantaged

**Table 9:** Multilevel logistic regression of active asthma

	*Model 1*					*Model 3*				
Covariates	*Est.*	*SE*	*p*	*[95%*	*CI]*	*Est.*	*SE*	*p*	*[95%*	*CI]*
Within-cluster										
Age	–	–	–	–	–	− 1.316	0.044	< 0.0001	[− 1.401	− 1.230]
Age^2^	–	–	–	–	–	− 0.291	0.026	< 0.0001	[−0.342	− 0.239]
Male	–	–	–	–	–	0.192	0.046	< 0.0001	[0.102	0.283]
Female	–	–	–	–	–	0.0	–	–	–	–
Smoker	–	–	–	–	–	− 0.324	0.071	< 0.0001	[− 0.462	−0.185]
Ex-smoker	–	–	–	–	–	−0.175	0.065	0.007	[−0.302	−0.049]
Never smoked	–	–	–	–	–	0.0	–	–	–	–
Indigenous Australian	–	–	–	–	–	0.201	0.209	0.336	[−0.209	0.612]
Non- Indigenous Australian	–	–	–	–	–	0.0	–	–	–	–
Unmarried	–	–	–	–	–	0.063	0.080	0.426	[−0.093	0.220]
Married	–	–	–	–	–	0.0	–	–	–	–
N-Comorbidities	–	–	–	–	–	1.741	0.035	< 0.0001	[1.672	1.811]
Between-cluster										
IRSD (Q4)	−0.091	0.077	0.241	[−0.242	0.061]	0.361	0.093	< 0.0001	[0.179	0.544]
IRSD (Q3)	−0.215	0.056	< 0.0001	[−0.324	−0.107]	0.055	0.071	0.443	[−0.085	0.194]
IRSD (Q2)	−0.135	0.047	0.004	[−0.228	−0.042]	− 0.020	0.064	0.759	[−0.145	0.105]
IRSD (Q1)	0.0	–	–	–	–	0.0	–	–	–	–

Notes – Age: age in years, standardised; Age^2^: Age*Age. Smoker: daily, weekly or irregular smoker; Ex-smoker: does not smoke now, but has smoked ≥ 100 cigarettes (or equivalent) in life time; Never smoked: does not smoke now, and has smoked < 100 cigarettes (or equivalent) in life time; Unmarried: never married, divorced, separated or widowed; Married: registered or de facto; N-Comorbidities: Number of comorbid conditions; IRSD: Index of Relative Social Disadvantage (ABS 2011); Q1-Q4: National quartile of IRSD (determined using cut-points for quartiles within the Australia-wide distribution of scores at SA1 level) where Q1: most disadvantaged, Q4: least disadvantaged

For asthma and area-level SES relationships, the unadjusted model resulted in a non-linear negative trend, whereby the comparison of the least (Q4) to the most (Q1) disadvantaged quartile groups was not statistically significant as might be expected from previous studies (Table 9). As shown in the model adjusting for individual-level covariates and numbers of comorbid conditions, this negative trend was reversed when comparing the fourth and third quartile groups (Q4, Q3) to the most disadvantaged (Q1) group. Only the comparison of the least (Q4) to the most (Q1) disadvantaged quartile groups was statistically significant, with the prevalence of asthma 1.44 times greater in the least disadvantaged SA1s (Q4) compared to the most disadvantaged SA1s (OR = 1.435; 95% CI: 1.196–1.723) (Table 9). The adjusted model confirmed the importance of individual-level covariates other than ATSI and marital status, particularly the number of comorbid conditions which exerted a strong influence and was responsible for the shift from the negative non-linear trend observed in the non-adjusted model.

### Spatial patterns of COPD, asthma and SEIFA - index of relative social disadvantage

**Fig. 3:**
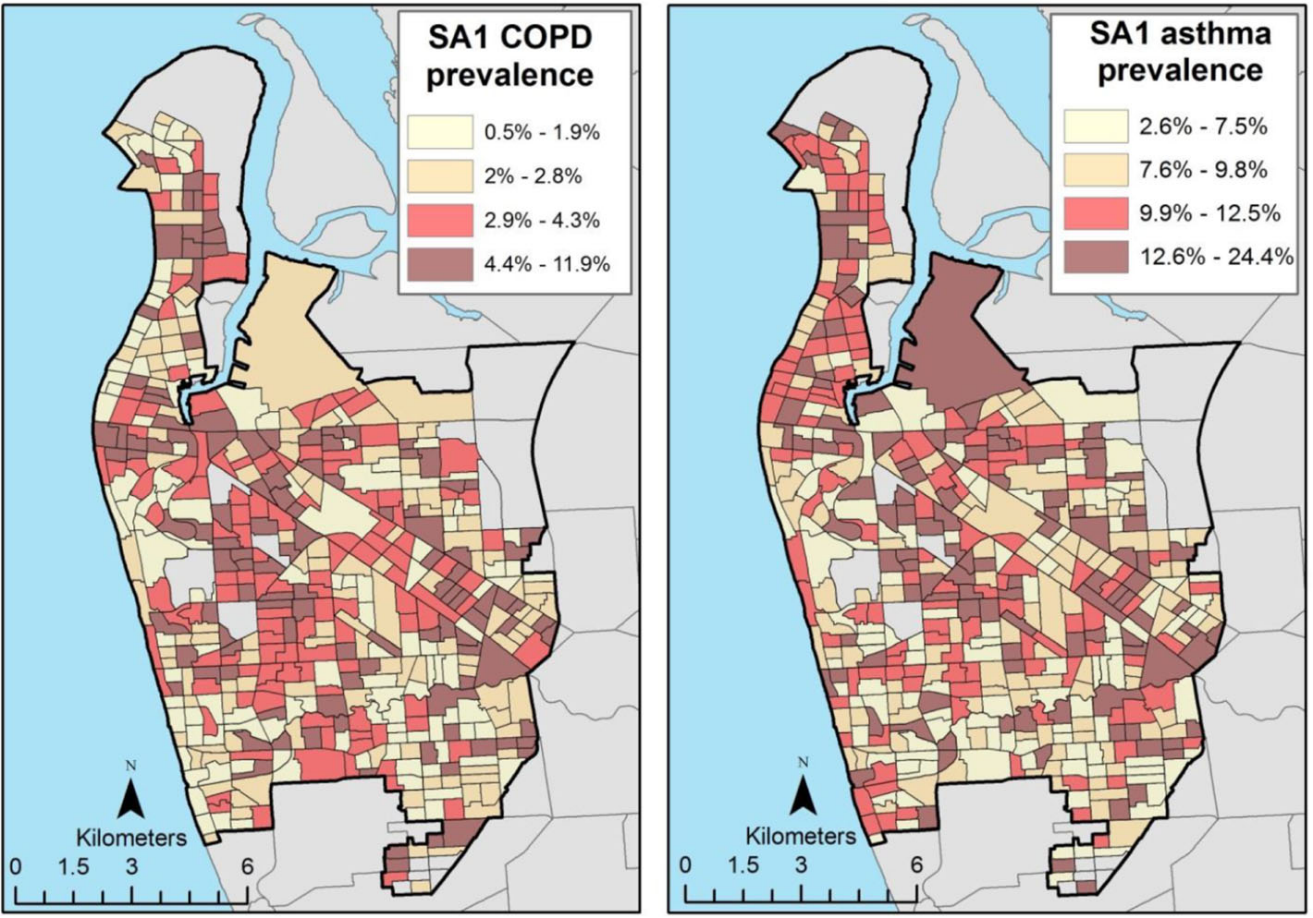
Estimated prevalence of COPD and asthma by SA1

Both COPD and asthma cases were not randomly distributed throughout the study region, but exhibited degrees of geographic aggregation. For COPD, low prevalence values were observed for western coastal areas, with some pockets of high prevalence. High prevalence areas were observed for northern regions, towards the east to the periphery of the central business district, and in the south, close to the airport (Fig. 3). For asthma diagnoses, areas with high prevalence values were observed in the northern region and the northwest-southeast axis of the study region. The southwest, south and southeast regions had low prevalence values in general, but these were punctuated with pockets of high prevalence (Fig. 3).

**Fig. 4:**
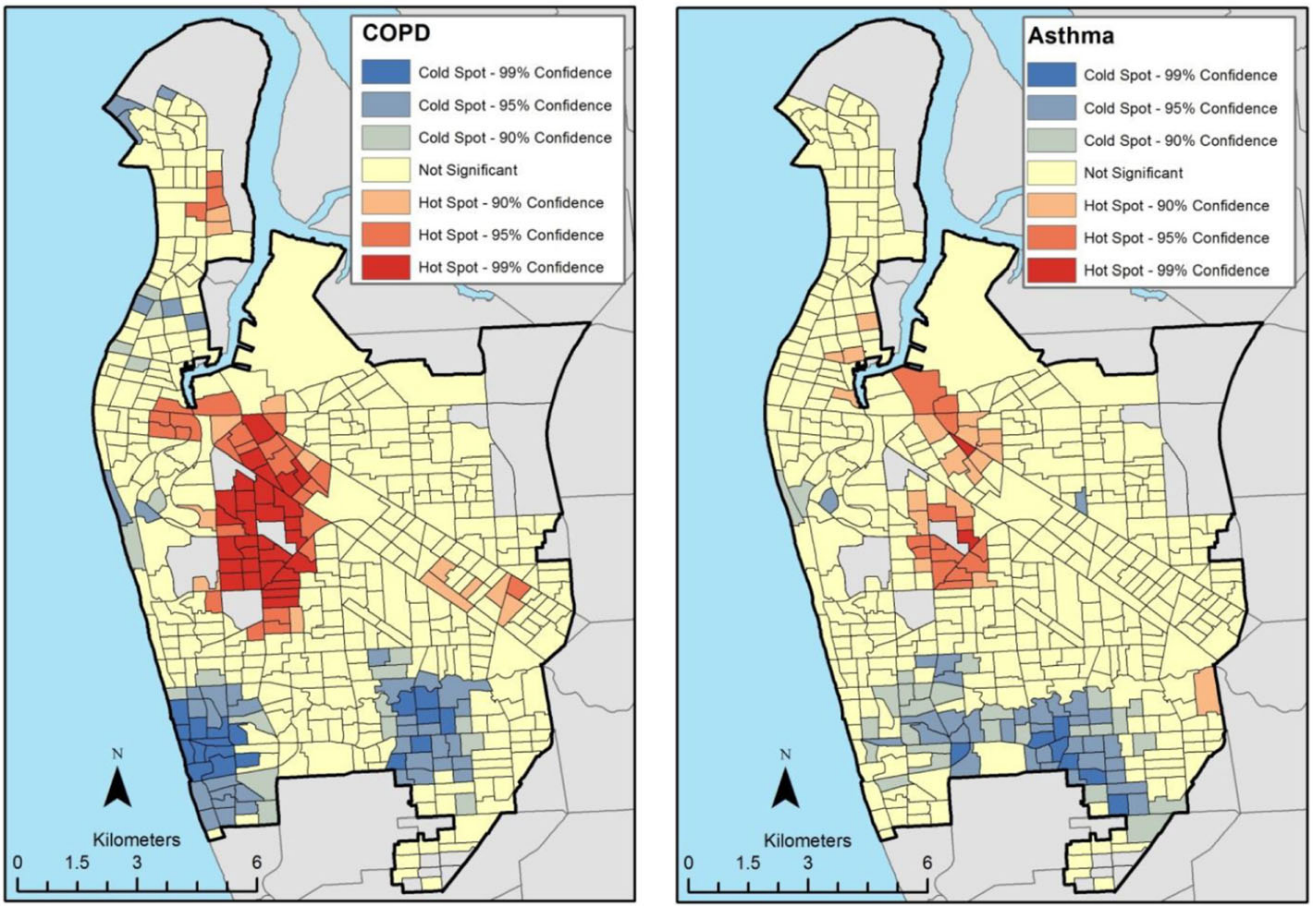
Clustering of COPD and Asthma cases (Hot and cold spots)

Both COPD and asthma prevalence values exhibited global and local spatial relationships. The Moran’s I statistic for COPD (*I* = 0.033, *p* = 0.002) indicated a positive and statistically significant spatial autocorrelation (small-scale variation) with the clustering of one large hot spot, two very small-size hot spots and two relatively medium-size cold spots (Fig. 4). For asthma prevalence, the Moran’s I statistic (*I* = 0.0178, *p* = 0.076) indicated positive global spatial relationship, but at the borderline of statistical significance. The clustering of high values (one medium-size hot spot) and low values (one relatively large cold spot) are illustrated in Fig. 4.

**Fig. 5:**
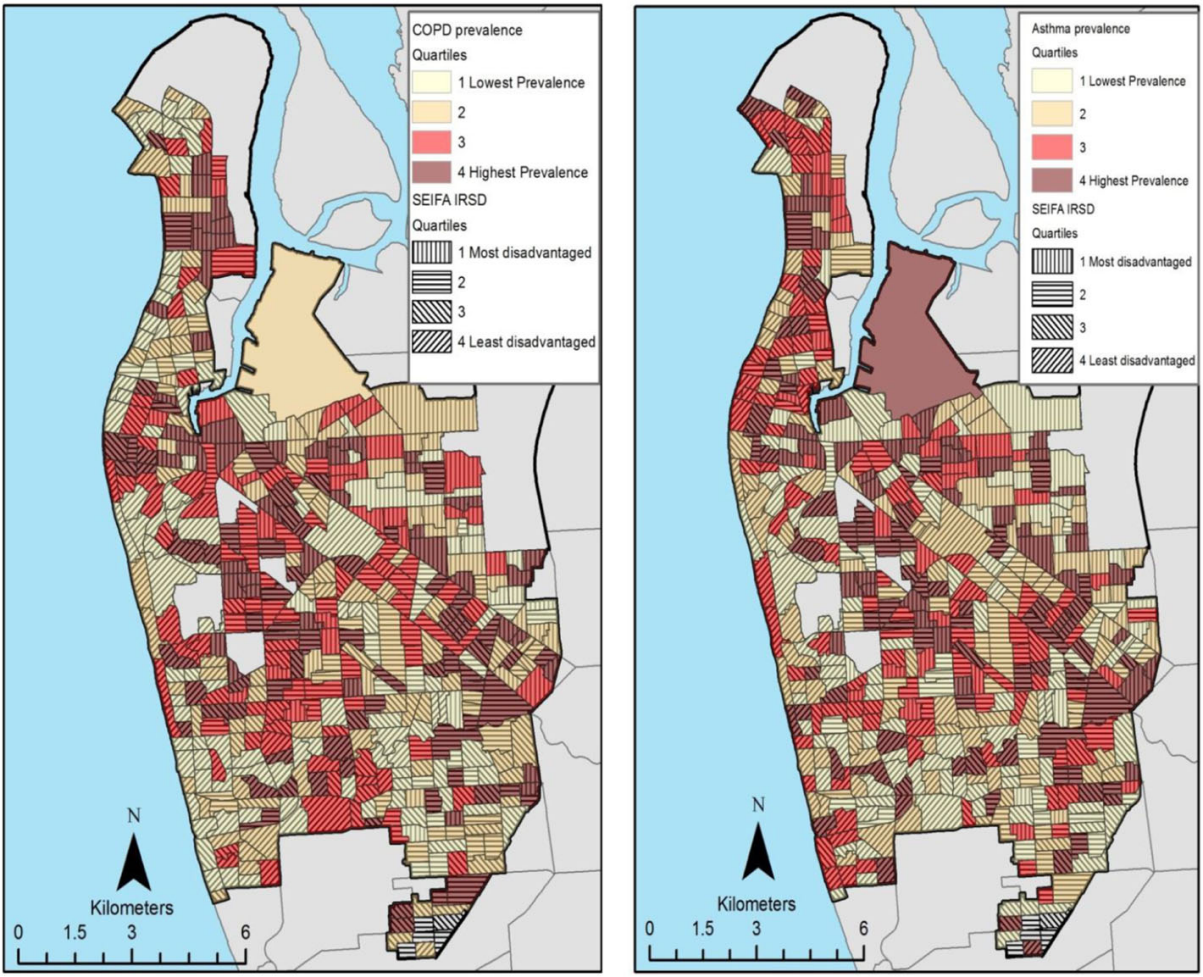
Choropleth maps overlaying COPD and asthma prevalence rates with relative socio-economic disadvantage

Geographic patterns of the index of relative social disadvantage (SEIFA-IRSD) highlighted the coastal west and southwest region with relatively well-off areas with social disadvantage increasing towards the north, close to the peninsula and towards both the east and southeast regions (Fig. 5). Overlaying choropleth maps of COPD and asthma prevalence rates with relative socio-economic disadvantage (Fig. 5) confirmed the presence of relatively high prevalence rates of COPD and asthma in low SES areas, but did not show any specific trend as expected, on the basis of the inverse linear relationship previously described.

## Discussion

This analysis of GP-based data examined patients’ respiratory conditions and showcased the practical utility of these data for research to improve practitioners’ understandings of chronic disease, and the individual and geographic factors related to chronic disease and the potential application for improving health care practice and patient outcomes [26, 39]. Benchmarked with age-specific 2014–15 NHS estimates, GP-data revealed lower estimates for COPD up to 64 years, then higher estimates for age 65 and over. These high-risk age groups are more likely to be captured by GP practices than NHS data, as GPs will consider a diagnosis of COPD for those patients with a history of exposure risk factors and comorbid conditions as recommended by the guidelines [40, 41]. For other patients, symptoms may appear non-specific and may be missed, resulting in under-diagnosis of COPD [42, 43]. In relation to asthma, the GP data revealed higher prevalence values in the 15–25 and 75+ age groups compared with the NHS-based estimates. Indeed, patients with childhood asthma may become asymptomatic, no longer require treatment, or be missed at diagnosis. However, those that might outgrow asthma could still develop asthma later on in their lives, being vulnerable.

These discrepancies may indicate a better understanding of the real-world health status and outcomes that routine GP-practice based data offer as opposed to self-reported survey-based data. Knowledge of practice-based versus population discrepancies may also serve to raise GP awareness and vigilance when assessing respiratory symptoms, relevant not only to early detection, but to avoiding delayed detection and missed opportunities to prevent worsening disease outcomes [43, 44]. This may be especially important in age groups for whom the lower prevalence estimates of GP-based data compared with the NHS could indicate possible under-diagnosis. Finally, these research findings stand to assist GPs in continuing education for better management of patients’ multiple diseases and conditions, and in developing accountability benchmarks that could improve patient care – provided these benchmarks focus on getting the best outcome for the patient, not simply meeting certain imposed targets for service delivery [23, 28, 45]. Although this is an idealistic expression of the concept of putting data back into GP-practices to improve patients’ outcomes, given substantial differences between guideline-based and GP-recorded management of asthma and COPD, this feedback of practice-relevant information could be a critical component of uptake of guidelines into daily practice and enhancement of support strategies available at multiple levels [22].

Other lessons derived from this GP-based data analysis pertain to the co-occurrence of COPD and asthma with other chronic conditions, especially COPD-osteoarthritis/osteoporosis and COPD-mental health (similarly, asthma-osteoarthritis/osteoporosis and asthma-mental health) pairs. High rates of co-occurrence were observed among patients aged 55 and over, highlighting the extent of age-related burden managed by GPs in routine practice [46, 47]. High rates of co-occurrence were reported by Britt and Miller in 2013 with COPD being associated with two or more other chronic diseases in about 87.4% of patients aged 65 years and over [27]. Other studies have documented the systemic effects of COPD on mental health and the management of depressive symptoms or severe osteoarthritis in patients with COPD [48, 49].

The geographic patterning of COPD and asthma prevalence estimates with area-level SES has previously been reported [50]. SES (at both individual and area levels) has been linked to various health outcomes including asthma and COPD, with lower SES being associated with higher rates of morbidity and mortality [14, 50–55]. In this study, there was a clear negative trend along the areal SES continuum for COPD with clustering most likely being driven by the presence of health care services (hospitals and GP centres) [12, 56]. This may be due to the population in the study area being mostly multicultural and within the lower range of SES, except the west coastal region. Moreover, moderate to severe COPD patients may choose to live closer to GP centres and local hospitals to improve access time.

The most obvious limitation of this study is the lack of GP data from other sub-regions in Western Adelaide plus the wider Adelaide metropolitan area, especially from areas with higher SES and including Adelaide CBD. This would have allowed for comparisons of areas with greater variability in SES and a greater extent of geographic distributions in prevalence estimates, given that allied health and other health services tend to be concentrated in inner urban areas and the CBD. A further limitation is a lack of data on clinical variables (e.g. spirometry) related to COPD and asthma severity and management, as well as the extent of referrals to specialized services including community respiratory services and rehabilitation centres.

Finally, the samples of practices and patients may not be representative of Australia, therefore limiting the generalizability of findings and restricting inferences to the Adelaide metropolitan region. However, lessons learnt might more likely have relevance to other metropolitan regions in other states or territories in Australia [57]. To some extent these lessons could be generalised, as most GP practices use computers and majority code diagnoses using a range of coding systems. All across Australia, via primary health networks, efforts have been applied at local level to improve the quality of GP recorded health data, as exemplified by the BEACH program data [27] and Magnet research platform data [28].

Further research is indicated to: clarify reasons for under-diagnosis and/or over-diagnosis of chronic respiratory conditions in primary care settings [25]; target disparities in chronic respiratory diseases and potential correlates, such as psychosocial stressors and built environment factors [58–60]; and most importantly, highlight the net benefits of early detection on other patients’ outcomes such as quality of life, and other comorbid conditions [41, 57, 59].

## Conclusion

GP-based research databases are vital to achieving improved understandings of factors that shape patient outcomes including COPD and asthma, the burden of disease and comorbid conditions, and levels of disease management and quality of health care achieved. Although based on a limited number of GP practices in a geographically specific area, our results highlight the practical utility of routine GP-based data for quality research that can guide GPs with regard to interventions including targeted screening and management to improve patient outcomes. Observed spatial patterns suggest that efforts to address inequalities in COPD and asthma, and their comorbid conditions, should account for the context of both practices and patient local contexts as well as social and environmental barriers to change. This may require investment in continuing medical education for professionals, not only to increase their awareness and skills in COPD and asthma management and control including early diagnosis, but also to improve clinical coding for these conditions in GP practices.

## Abbreviations

ABS: Australian Bureau of Statistics
ACO: Asthma-COPD Overlap syndrome
AIHW: Australian Institute of Health and Welfare
BEACH: Bettering the Evaluation and Care of Health
CBD: Central Business District
CI: Confidence Interval
COPD: Chronic Obstructive Pulmonary Disease
GINA: Global Initiative for Asthma
GOLD: Global Initiative for Chronic Obstructive Lung Disease
GP: General Practitioners
IRSD: Index of Relative Socio-economic Disadvantage
MAGNET: Melbourne East Monash General Practice Database
Meteor: Metadata Online Registry
NHS: National Health Survey
OR: Odds Ratio
PHA: Practice Health Atlas
Q1-Q4: Quartiles
RACGP: Royal Australian College of General Practitioners
SA1: Statistical Area 1
SEIFA: Socio-Economic Index of Areas
SES: Socio-Economic Status
SPR: Standardised Prevalence Ratio
